# Green Extraction of *Citrus sinensis* (L.) Osbeck Peel Essential Oil and Its Impact on Antioxidant and Antimicrobial Activity: Supported by Molecular Docking and DFT Analysis

**DOI:** 10.1002/fsn3.71935

**Published:** 2026-06-11

**Authors:** Nehha Nadeem, Ahmed B. M. Ibrahim, Muhammad Adnan Ayub, Khayala Mammadova, Muhammad Ijaz, Mohamed A. Habib, Mohamed Fawzy Ramadan, Nasrin Choobkar

**Affiliations:** ^1^ Department of Chemistry University of Sahiwal Sahiwal Pakistan; ^2^ Department of Chemistry College of Science, Imam Mohammad Ibn Saud Islamic University (IMSIU) Riyadh Saudi Arabia; ^3^ Medical and Biological Physics Department Azerbaijan Medical University Baku Azerbaijan; ^4^ Department of Clinical Nutrition, Faculty of Applied Medical Sciences Umm Al‐Qura University Makkah Saudi Arabia; ^5^ Plant Biotechnology Research Center, Ker.C. Islamic Azad University Kermanshah Iran

**Keywords:** biological activities, GC–MS analysis, superheated steam extraction, synergistic effect frontier molecular orbitals

## Abstract

*Citrus sinensis* (L.) Osbeck ( *C. sinensis* ) peel is a rich source of essential oil (EO) widely used in pharmaceutical, food, and aroma industries; however, effective extraction strategies and molecular insights remain unexplored. This study presents superheated steam extraction (SHSE) as a novel green approach for the *C. sinensis* EO extraction and its comparison with conventional hydro and steam distillation methods. The chemical profile and bioactivities were analyzed and further strengthened by molecular docking and DFT studies. A maximum yield of 0.20% (w/w) was obtained through SHSE. Volatile composition, studied by GC–MS, identified d‐limonene (8.84% to 30.72%) as the primary compound. Several tests, including resazurin microtiter plate assay, agar well diffusion, and microdilution broth assay, were performed to evaluate the antimicrobial potential. The SHS extracted EO exhibited maximum antibacterial and antifungal activity. Antioxidant properties of the extracted EOs were determined by employing the DPPH assay, hydrogen peroxide scavenging assay, and the FRAP assay. Superheated steam extracted EO also revealed the highest antioxidant activity. Molecular docking was performed to evaluate the ligand–protein interactions. All compounds showed lower binding energies; among them, four compounds, d‐limonene, *trans*‐carvyl acetate, β‐terpinyl acetate, and α‐himachalene, were found as significant binders to the targeted antioxidant and antimicrobial proteins. DFT studies were performed at the B3LYP/6–31G(d,p) level to elucidate the compound's antioxidant mechanism of action. DFT predicted β‐terpinyl acetate as the most effective antioxidant candidate, attributed to its lowest energy gap (6.7616 eV), highest chemical softness, and lowest chemical hardness. Overall, it is concluded that SHSE gave the highest EO yield while biological assays provided the synergistic performance of EO components which is justified by combining with computational analysis.

## Graphical abstract

Superheated steam extraction of *Citrus sinensis* peel essential oil produced the highest yield and enhanced antioxidant and antimicrobial activities compared with conventional methods. GC–MS identified d‐limonene as the major compound, while molecular docking and DFT analyses confirmed strong bioactive interactions and highlighted β‐terpinyl acetate as a promising antioxidant candidate.

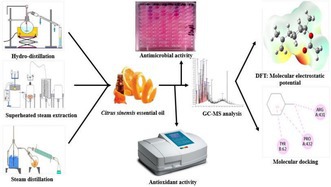

## 1 Introduction

Today, foodborne illnesses are a significant issue affecting millions of people worldwide. These problems are caused by the intake of contaminated food or water, containing some pathogenic bacteria, viruses, fungi, or other protozoa (Ayub, Goksen, et al. 2023). A previous study reported that *C. jejuni* , *S. enterica, S. aureus, E. coli, and L. monocytogenes* are particularly associated with foodborne diseases (Ayub, Choobkar, et al. 2023). Free radicals are generated when electrons are split off during metabolic reactions, and this process may lead to diseases such as oxidative stress, neurodegeneration, and diabetes (Zymone et al. 2022). These illnesses can be controlled by antioxidants, which neutralize them. Antioxidants are divided into two main categories: natural and synthetic preservatives. Synthetic preservatives exhibit both antioxidant and antimicrobial activities; however, they may cause intoxication, allergies, and degenerative diseases (Laranjo et al. 2017). Therefore, scientists shifted their focus to natural preservatives.

Essential oils are the natural antioxidants used to mitigate the effects of free radicals (Myszka et al. 2019). Essential oils, often termed *plant essences*, are complex blends of volatile compounds synthesized by various living organisms, particularly plants. They serve as secondary metabolites of plants, contributing to their unique aroma and properties due to their significant chemical composition. They are used in the aroma, pharmaceutical, food, and flavor industries. Moreover, they possess substantial anti‐inflammatory, antioxidant, antimicrobial, anticancer, and cytotoxic potential (Ayub et al. 2018). They are renowned for their intense nature and diverse fragrances, and they enrich our lives with their versatility and health benefits (Hüsnü et al. 2007; Manion and Widder 2017).

The Citrus genus is a part of the *Rutaceae* family, which consists of approximately 1300 species (Flamini et al. 2003). *C. sinensis* originates from Asia and has subsequently spread worldwide. *C. sinensis*, which is a perennial flowering tree, typically grows to a height of 9–10 m and has prominent spines on its limbs. Leaf assembly is discontinuous, with petioles that are narrow and wing‐shaped, having a diameter of 3–5 mm and a length of 6.5–15 cm. Its blades have a shape that can be abstruse, oblong to oval, and bluntly curved, and possess a strong, peculiar smell owing to the presence of EO (Goldhamer et al. 2012).

Among plant essential oils (PEOs), citrus EOs have garnered considerable attention due to their versatile properties, including broad‐spectrum insecticidal, antibacterial, and antifungal effects. Additionally, CEOs offer high yields of essential oils along with pleasant scents and flavors. Moreover, they find widespread applications in food preparations, packaging, and preservation, playing a vital role in attaining good quality and safety of food (Calo et al. 2015).

The EOs can be extracted by processing the plant material through HD, SD, and superheated steam extraction (SHSE). Hydro distillation is an ancient method to extract EOs from plant materials. Although it is a straightforward method, it has certain drawbacks, including slow extractions, possible chemical alterations due to water exposure, and more consumption of both fuel and water. Steam distillation is more reliable and convenient than HD. Choosing steam over water often makes it possible to collect more EO yield from this method. On the other hand, it also has some disadvantages, like a lengthy extraction process, potential contamination from steam, and reduced accuracy (Charles and Simon 1990; Fatima et al. 2025).

The SHSE is an emerging EO extraction technique that utilizes high‐temperature steam ranging from 101°C to 180°C to ensure efficient extraction of EO. SHSE produces the most effective yield due to the steam's lower viscosity, polarization, higher penetration power, and larger kinetic energy content. Due to its high temperature, SHS possesses additional energy and penetration capability compared to conventional steam, potentially enhancing the extraction rate. The rising steam temperature decreases the dielectric constant while improving diffusion rates due to reduced viscosity and surface tension (Ayub, Goksen, et al. 2023; Ayub et al. 2018). This technique has many benefits over conventional methods, including improved heat transmission, increased total extraction yield, and controllable oxygen levels (Anjam et al. 2025). On the other hand, the high temperature of SHS may degrade the heat sensitive compounds.

It is common to use structure‐based virtual screening, such as molecular docking, in drug design because it is efficient and leads to lower costs as compared to experiments. This approach helps predict how strong and in which direction a drug candidate will bind to the active site of the target protein. Additionally, it enables the screening of many possibilities and helps identify specific lead compounds. The density functional theory calculations reveal key information about the electronic structure, geometries, and chemical behavior of molecular systems. Predicting reactivity and stability, DFT is crucial for determining possible reactive sites and understanding charge distribution within molecules. Moreover, it facilitates the examination of frontier molecular orbital energy levels, which affect molecular interactions. These insights from quantum mechanics greatly aid in the logical development and refinement of molecules that resemble drugs (Anjam et al. 2025; Zochedh et al. 2022).

Previous studies have shown that extraction techniques including hydro distillation and steam distillation have been commonly employed for the *C. sinensis* essential oil extraction (Hacib et al. 2024) (Bozova et al. 2024) (Dewi et al. 2018). However, the SHSE technique with its extraction conditions has not yet been applied to extract the essential oil from *C. sinensis* . By notifying this, the present work applies the SHSE technique, using its specific extraction parameters like temperature, pressure, and flow rate, to extract the *C. sinensis* essential oil and compares its efficiency with conventional extraction techniques. Both the qualitative and quantitative analysis of CSEO's chemical composition were performed by GCMS analysis. Moreover, the synergistic biological potential of CSEO was determined by applying different antioxidant and antimicrobial assays. Additionally, molecular docking and DFT give information about the targeted ligand–protein interactions and electronic behavior of selected chemical compounds.

## 2 Methodology

### 2.1 Plant Material Collection

Fresh *Citrus sinensis* (L.) Osbeck ( *C. sinensis* ) peels were collected from Sahiwal (30.6^o^ north latitude and 73.1^o^ longitude) in central Pakistan, demonstrating a proactive approach to resource utilization. The plant material was taxonomically identified by Dr. Fahim Arshad, Department of Botany, University of Okara, Pakistan. A voucher specimen (No. BGPU2712012020) was prepared and deposited in the Botanical Herbarium of the Department of Botany, University of Okara, for future reference. The plant material was dried under a shed until constant weight, ground into a 60‐mesh size for further processing. Overall experimental details are presented in the graphical abstract.

### 2.2 Extraction Methods

*C. sinensis* EO was effectively extracted by using three different types of extraction techniques, including HD, SD, and SHSE, each contributing special characteristics to the resultant composition. The following formula was used to calculate the percentage of EO yield:

$$\%\mathrm{age}\;\mathrm{EO}\;\text{yield}=\frac{\mathrm{EOs}\;\text{in grams}}{\text{Sample weight in}\;\left(g\right)}\times 100$$

#### 2.2.1 Hydro‐Distillation

A Clevenger‐type apparatus was used for hydro‐distillation, which contains conventional components such as a 5000 mL round‐bottom flask, a heating mantle, a Dean‐Stark trap, condensers, and a separating funnel. In a round‐bottom flask, 300 g of fresh plant material was mixed with 5000 mL of distilled water. The water boiled due to the heat provided by the heating mantle, which carried the steam mixture having high‐boiling water and *C. sinensis* EO. The oily layer was separated by using a separating funnel (Fatima et al. 2025).

#### 2.2.2 Steam Distillation

The SD setup comprises a biomass flask containing plant material, a condenser, a boiling setup, a thermometer, and a separating funnel. The 300 g of *C. sinensis* peels were placed in the plant biomass flask above the circular bottom flask. The steam produced by boiling water interacts with the plant material in the biomass flask, resulting in the extraction of essential oil (EO) and water vapors. A condenser was utilized to cool the vapors and condense them into EO and hydrosol. The difference in density between the hydrosol and the oil led them to separate in the collection vessel. Afterward, the EO was separated, mixed with anhydrous sodium sulfate, filtered with a microfilter, and stored in a glass vial. The extraction was repeated three times (Ayub, Choobkar, et al. 2023).

#### 2.2.3 Superheated Steam Extraction

The apparatus of the SHSE technique consists of a stainless‐steel extraction vessel, an SHS generator, a condenser unit, and a hydrosol collection tank. The plant biomass was uniformly dried to a constant weight to ensure consistent heat and mass transfer. A total of 300 g of dried plant material was placed in the extraction chamber and subjected to SHS at 150°C and 60 psi for 3 h. The flow rate was maintained at 60 mL/min throughout the extraction process. The system was operated in a closed configuration to minimize oxygen exposure and prevent oxidative degradation of volatile compounds. Moreover, the temperature profile was continuously monitored and regulated using thermocouples installed at the inlet and outlet of the superheated steam generator. After extraction, the moisture in the EO was removed with anhydrous sodium sulfate (0.25 to 0.5 g) and filtered through a microfilter. A microfilter was applied to purify the EO, which was then placed in the amber glass vial. Reliability was secured by performing the extraction process three times (Ayub, Iram, et al. 2024).

### 2.3 Gas Chromatography–Mass Spectrometry (GC–MS) Analysis

Gas chromatography–mass spectrometry (GC–MS) was used to analyze the chemical profile of each EO obtained via various extraction processes. The Shimadzu GC–MS‐QP 2010 system was equipped with an MS detector and the DB‐5 capillary column (50 m x 0.25 mm, 0.25 μm film thickness). Using a syringe, 2 μL of EO diluted 1:100 in ethanol was injected into the system for analysis. To perform the injection, a split ratio of 1:50 was used in split mode. The pressure of the column head was kept between 200 kPa and 300 kPa. The temperature of the injector was maintained at 200°C. The temperature of the GC column was initially set at 60°C for 3 min, then gradually increased to 240°C at a rate of 24°C/min and maintained for 10 min. Helium gas served as a carrier gas, maintaining a flow rate of 1.0 mL/min.

The mass spectrometer transfer line and ion source temperatures were maintained at 220°C and 230°C, respectively. MS detection was carried out using an electron ionization energy of 70 eV. Before performing the analysis, PFTBA, Perfluorotributylamine, was employed to tune and calibrate the MS system for ensuring the necessary mass axis calibration and sensitivity. Multipoint external standards were used to construct the calibration curves of the target analytes in a concentration range of 10 to 1000 μg/mL. Peak area normalization was used for the semi‐quantitative analysis. The retention index for all compounds was determined by comparing them to n‐alkanes (C_9_ to C_24_). The data from the retention index and mass spectrum were compared to the information available in the NIST library and Pherobase mass spectral database. The chemicals were confirmed through co‐injection of the genuine standards. It was done to conduct a quantitative examination of the essential oil components (Anjam et al. 2025). The given equation was employed to determine the RF values:

$$\mathrm{RFc}=\left(\frac{\mathrm{Ac}}{\mathrm{Ais}}\right)/\left(\frac{\mathrm{Cc}}{\mathrm{Cis}}\right)$$

Response factor of the EOs component is denoted by RF.

Ac = Peak area of the EO component.

A_is_ = Peak area of the internal standard.

Cc = Concentrations of the components of the EO.

C_is_ = Concentrations of the internal standard.

Applying the RFs, the percentage of each component of EO was determined as follows:

$$\text{Corrected area}=\frac{{\text{Compound}}^{'}s\;\text{peak area}}{{\text{Compound}}^{'}s\;\text{response factor}}$$

$$\text{Percentage}\;\left(\%\right)=\left(\frac{{\text{Compound}}^{'}s\;\text{corrected area}}{\text{total of corrected areas}}\right)\times 100$$

### 2.4 Antimicrobial Activity

The essential oils (EOs) were tested for antimicrobial activity against a variety of microorganisms, including the bacterial strains *Escherichia coli* (ATCC 25922), *Staphylococcus aureus* (ATCC 25923), and fungal strains including *Fusarium solani* (ATCC 36031) and *Aspergillus niger* (ATCC 16404).

#### 2.4.1 Agar Well Diffusion Method

The agar well diffusion method was employed to assess the EOs antibacterial and antifungal potential. After overnight culturing, microbial strains were transferred to a 25 mL growth medium solution. The mixture was then put into medium‐sized Petri plates and left to solidify at room temperature. Wells were then made with a sterilized cork borer (having a diameter of 6 mm). These agar wells were loaded with 10 mg of EO and the standard drug (1 mg/mL), which was ampicillin for bacterial strains and fluconazole for fungal strains. Bacteria were incubated in Petri dishes at 37°C for 24 h. Following the incubation period, the inhibitory zones (mm) were measured to evaluate the antibacterial and antifungal potential (Ayub et al. 2025).

#### 2.4.2 Resazurin Micro‐Titer Plate Assay

The resazurin microtiter plate test was used to determine the minimum inhibitory concentration (MIC) of EOs against various bacterial strains. For the preparation of the sample solution, 10 mg of EO was dissolved in 1 mL of 10% DMSO. A resazurin indicator solution was prepared by dissolving 27 mg of resazurin in 4 mL of deionized water. The analyte solution (100 μL) and positive control (1 mg/mL in 10% DMSO) were pipetted into the first row of 96‐well plates. Then, 50 μL of nutrient‐rich broth was added to each well, except those in the first row. Two‐fold serial dilutions were made with 50 μL of material in each well until each well contained 50 μL of the analyte mixture. Then, 30 μL of iso‐sensitized broth (3.3× strength), 10 microliters of resazurin solution, and 10 μL of microbial culture (5 × 10^5^ colony‐forming units/mL) were added to each well. The MIC values were visually evaluated after incubation at 37°C for 24 h. A shift in color from purple to pink or colorless indicated the bacterial proliferation. The lowest concentration at which the color shift occurred was used to determine the MIC values (Hussain et al. 2010).

#### 2.4.3 Micro‐Dilution Broth Assay

The MIC of the EO solutions against different kinds of fungi was estimated using the microdilution broth method. About 10 mg of essential oil was mixed with 1 mL of 10% DMSO to make a sample solution, and 1 mL of standard antibiotic fluconazole was mixed with 1 mL of a 10% DMSO solution to make the solution of standard antibiotic fluconazole. A 100‐μl sample and standard volume were added to the first row of the 96‐well plates. Then, 50 μL of Sabouraud dextrose was added to each well, except those in the first row. After conducting the two‐fold serial dilution procedure, every well contained 50 μL of the sample solution. This was followed by the addition of 130 μL of the Sabouraud solution and 20 μL of the microbe suspension (at a concentration of 5 × 10^5^ colony‐forming units/mL) to each well. Afterwards, the plates were put in the incubator at 30 degrees Celsius for 48 h. The MIC value, which corresponds to the lowest possible concentration that completely prevents fungal growth, was visually determined (Dabur et al. 2004).

### 2.5 Antioxidant Activity

#### 2.5.1 DPPH Free Radical‐Scavenging Activity

In the DPPH test, 1 mL of a DPPH solution with a concentration of 0.09 mM was mixed with 2.5 mL of 100 mg/mL of EO in ethanol. After that, the mixture was made up to 4 mL using methanol and kept in the dark for about an hour at room temperature. Gallic acid was used as a standard. The absorbance measurements have been taken at 515 nm (Mensor et al. 2001). The % DPPH scavenging activity was determined accordingly:

$$\%\mathrm{age}\;\text{DPPH}-\text{FRSA}=\;\frac{\mathrm{Abs}\;\text{sample}}{\mathrm{Abs}\;\text{control}}\times 100$$

#### 2.5.2 Hydrogen Peroxide (HP) Scavenging Activity

The HP scavenging activity of essential oils has been assessed using a spectrophotometric method. A 2 mM hydrogen peroxide solution was produced in a phosphate buffer (0.17 M, pH 7.4), and ascorbic acid (100 mg/L) was added. A volume of 0.6 mL of 100 mg/mL of EO in ethanol was mixed with 600 μL of hydrogen peroxide. After a 10‐min incubation, the absorbance at 230 nm was measured to determine the percentage of scavenging activity against hydrogen peroxide. A similar process was repeated for the H_2_O_2_ activity of the standard, ascorbic acid (100 mg/L) (Ayub et al. 2018).

$$\%\mathrm{age}\;\text{of hydrogen peroxide scavenged}=\frac{A_{0}-A_{1}}{A_{0}}\times 100$$

Here, A_0_ signifies the absorbance of the control, and A_1_ signifies the absorbance of the sample.

#### 2.5.3 Reducing Power Ability (RPA)

The Assessment of RPA Involved Mixing 1 mL of Either an EO Sample (100 mg/mL of EO in Ethanol) or a Standard Solution of Gallic Acid (25 to 100 mg/L) With 2.5 mL of 0.2 M Phosphate Buffer and 2.5 mL of 1% Potassium Ferricyanide. The Mixture Underwent a 25‐Min Incubation at 50°C Using Water. Subsequently, 2.5 mL of 10% Trichloroacetic Acid Was Introduced to the Mixture and Centrifuged at 1000 Rpm for 10 Min. Following That, the Resultant Solution (2.5 mL) Was Mixed With 0.5 mL of 0.1% FeCl3 and 2.5 mL of Distilled Water. The Absorbance at the Wavelength of 710 Nm Was Determined After 30 Min of Incubation at Room Temperature. Gallic Acid (0 to 100 mg/mL) Calibration Curve (y = 0.021x—0.0151, R^2^ = 0.99) Was Employed to Determine the Total Antioxidant Content (Habila et al. 2010).

### 2.6 Molecular Docking Analysis

The AutoDock Vina program was utilized for molecular docking, a virtual screening method that involves four key stages: protein preparation, ligand preparation, carrying out molecular docking, and reviewing the results. In this study, six protein structures 1CEX, 1KZN, 2CDU, 2I80, 3NRZ, and 7BLY were acquired and subsequently refined using UCSF Chimera. Ligands, crystallographic solvents, and buffer ions were all removed as part of refining the structure. Proteins were then protonated and assigned appropriate charges. Ligands underwent systematic preparation: their structures were obtained from PubChem in SDF format, converted into 3D models using Chem3D, and subsequently hydrogenated using Chimera.

A docking grid box of 30 Å on each side was used. The space of the network was set to 1.0, and 100 different binding modes were examined. Both the box and its coordinates were placed to fit the locations of the active binding sites perfectly. The docking performance was determined by calculating the binding energy (ΔG) in kilocalories per mole (kcal/mol). The software Accelrys Discovery Studio 4.1 (Dassault Systems BIOVIA, San Diego, CA, USA) was additionally used to study protein–ligand interactions (Noshad et al. 2022). The coordinates and dimensions of the docking grid box size were carefully selected to align with the active binding site, as shown in Table 1.

**TABLE 1:** Visualization of molecular docking parameters and protein targets.

PDB ID	Grid box center coordinate	Grid box size	Conformers per ligand
1CEX	center_x = −4.29943 center_y = 58.9737 center_z = 24.6483	size_x = 44.5846 size_y = 41.4064 size_z = 44.6199	8
1KZN	center_x = 18.5011 center_y = 18.0805 center_z = 44.4733	size_x = 47.9895 size_y = 42.631 size_z = 43.6023	8
2CDU	center_x = 12.4986 center_y = 4.63003 center_z = 25.0597	size_x = 62.3101 size_y = 66.2377 size_z = 101.111	8
2I80	center_x = 17.5209 center_y = −1.1512 center_z = 17.9005	size_x = 77.3705 size_y = 61.7922 size_z = 82.1531	8
3NRZ	center_x = 61.3966 center_y = 6.50303 center_z = 32.6814	size_x = 139.963 size_y = 92.4907 size_z = 95.6164	8
7BLY	center_x = 24.8587 center_y = 48.1451 center_z = −13.5177	size_x = 53.8651 size_y = 46.9257 size_z = 46.7578	8

### 2.7 Density Functional Theory

All 3D structures were made with Chem3D software. To produce the input files, the GaussView 6.0 program was selected, and all calculations were carried out using the Gaussian 09 software. All studies were performed within the B3LYP/6–31G (d,p) theoretical framework, allowing for geometry optimization, energy band gap finding, analyzing global reactivity, studying molecular electrostatics, and identifying the HOMO and LUMO as the molecular frontiers (Anjam et al. 2025).

### 2.8 Statistical Analysis

All the experiments were performed three times, and the data were checked using one‐way ANOVA (STATISTICA 5.5 software). Statistical significance was determined at *p* < 0.05. Standard deviation and mean value were calculated by averaging the results from three measurements.

## 3 Results and Discussion

### 3.1 Essential Oil Yields

*C. sinensis* EOs were isolated using various extraction techniques, including HD, SD, and SHSE; EO yield comparison is stated in Table 2. Superheated steam extraction exhibited the highest essential oil yield (0.20%), compared to conventional approaches. It has been observed that different extraction methods, particularly those used for essential oil (EO) extraction, significantly influence the EO yield. It was also noticed that when SHS was exposed to plant material, it released the highest yield as compared to other techniques, as seen in *Anethum graveolens* EOs (5.08%) (Fatima et al. 2025). It was reported that EO yield depends on various factors, including plant maturity, location of the plant, plant's specific part, genotype, tapping method, extraction conditions, and nutritional status (Ayub, Ijaz, et al. 2024). It has been found that solvent‐free microwave extraction (SFME) yielded 0.39% and HD yielded 0.31% EO from *C. sinensis* peels (Ngan et al. 2020). Similarly, *C. sinensis* was reported to yield 1.9%, 3.01%, 2.89%, 3.67%, and 3.58% EO through the different extraction techniques such as HD, salt‐assisted extraction by hydro‐distillation (S‐HD), ultrasound‐assisted extraction by hydro‐distillation (US‐HD), enzyme‐assisted extraction by hydro‐distillation (E‐HD), and SFME, respectively (Taktak et al. 2021). Furthermore, a comparative study of the EO yields obtained by the HD method from five different citrus plants revealed that red grapefruit produced a maximum EO yield (2.47%), followed by lemon (2.26%), white grapefruit (2.12%), orange (1.08%), and pomelo (0.53%) (Manaila et al. 2016).

**TABLE 2:** Essential oil yields of *C. sinensis* peels by different extraction techniques.

Extraction methods	Percentage yield
Hydro‐distillation	0.13 ± 0.02^b^
Steam‐distillation	0.15 ± 0.01^b^
Superheated steam extraction	0.20 ± 0.03^a^

*Note:* Values represent the mean ± standard deviations calculated from three independent trials. Different letters in the superscripts express significant variances in the EOs extracted from *C. sinensis* peels using various extraction methods.

Superheated steam extraction yielded the highest quantity of EO, which may be attributed to the steam's lower viscosity and polarity, as well as its higher penetration power and greater kinetic energy content (Ayub, Goksen, et al. 2023). It has been declared that SHSE, under optimal thermal conditions of 150°C, enhances the EO yield from *Origanum micranthum* leaves (Özel et al. 2005). According to earlier research, citrus is a rich source of EOs, with yields varying among plant species and usually ranging between 0.1% and 2.0% (Alrasheid et al. 2019). Superheated steam, at high temperature, breaks the plant cell walls and causes the EO extraction (Ayub, Goksen, et al. 2023). Additionally, improved wetting of the material, combined with deeper penetration into the material particles, improves the diffusivity, which enhances the mass transfer rate, leading to greater extraction process efficiency (Raza et al. 2024). Moreover, the extraction time also impacts the yield of the EO (Fatima et al. 2025). Overall, the present findings suggest that SHSE is a superior method for achieving greater EO yield from *C. sinensis* compared to traditional methods, HD, and SD.

### 3.2 Chemical Composition of *C. sinensis* EO

GC–MS determined the chemical composition of the extracted EOs. Overall, four classes of EOs components, including monoterpenes hydrocarbons, oxygenated monoterpenes, sesquiterpenes, and oxygenated sesquiterpenes, were obtained. Some compounds of other groups were also obtained. Results are presented in Table 3. Overall, eleven monoterpene HCs were obtained, including β‐pinene, β‐phellandrene, d‐limonene, l‐limonene, p‐cymene, p‐menthane, santene, iso‐limonene, camphene, β‐ocimene, and α‐pinene. The major monoterpene HC was d‐limonene, 8.84% to 30.72%, while the remaining monoterpene HCs β‐pinene, β‐phellandrene, l‐limonene, p‐cymene, p‐menthane, santene, iso‐limonene, camphene, β‐ocimene, and α‐pinene, appeared with a concentration ranging between (1.56% to 2.46%), (2.46% to 2.81%), (0.1% to 2.68%), (0.44% to 0.66%), (3.56% to 5.94%), (1.08%), (2.7%), (1.81%), (1.65%), and (1.08%), respectively.

**TABLE 3:** Chemical composition of the extracted EOs by various extraction methods.

Sr. No	Component	RT^A^	RI^B^	% composition of essential oil			Method of identification
				HD	SD	SHSE	
Monoterpene hydrocarbons							
1	β‐Pinene	5.117	980	1.56 ± 0.01^b^		2.46 ± 0.05^a^	a, b
2	β‐Phellandrene	5.122	1030	2.81 ± 0.08^a^		2.46 ± 0.07^b^	a, b
3	d‐Limonene	5.614	1031	8.84 ± 0.1^c^	17.46 ± 0.15^b^	30.72 ± 0.20^a^	a, b
4	l‐Limonene	5.994	1032	0.1 ± 0.00^b^		2.68 ± 0.1^a^	a, b
5	p‐Cymene	6.406	1026	0.66 ± 0.01^a^		0.44 ± 0.01^b^	a, b
6	p‐menthane	6.823	978	3.56 ± 0.03^c^	5.94 ± 0.02^a^	4.49 ± 0.03^b^	a, b
7	Santene	7.647	1295	1.08 ± 0.02^a^			a, b
8	Iso limonene	8.626	983	2.7 ± 0.02^a^			a, b
9	Camphene	8.749	1090			1.81 ± 0.03^a^	a, b
10	β‐Ocimene	8.84	1035			1.65 ± 0.1^a^	a, b
11	α‐pinene	9.198	1045	1.08 ± 0.02^a^			a, b
Oxygenated monoterpene hydrocarbons							
12	Eucalyptol	6.101	1033	0.41 ± 0.01^a^			a, b
13	Linalool oxide	6.363	1025	0.43 ± 0.02^b^		1.80 ± 0.03^a^	a, b
14	Linalool	6.491	1098	3.92 ± 0.03^b^	7.59 ± 0.03^a^	2.18 ± 0.01^c^	a, b
15	Limonene Oxide	6.919	1070	6.13 ± 0.02^a^	2.56 ± 0.02^b^	1.53 ± 0.01^c^	a, b
16	Citronellal	6.989	1153			0.38 ± 0.01^a^	a, b
17	Camphor	7.064	1143	0.97 ± 0.01^a^			a, b
18	*trans*‐Isopiperitenol	7.12	1050	1.33 ± 0.01^b^		1.54 ± 0.02^a^	a, b
19	α‐Campholenal	7.192	1192	0.97 ± 0.01^a^		0.73 ± 0.01^b^	a, b
20	Carvomenthone	7.267	1023	1.7 ± 0.01^a^			a, b
21	Carvomenthenol	7.347	1189		2.98 ± 0.02^a^	0.5 ± 0.01^b^	a, b
22	Citral	7.353	1005			1.09 ± 0.02^a^	a, b
23	Geraniol	7.374	1255	3.31 ± 0.02^a^		0.17 ± 0.01^b^	a, b
24	Estragole	7.513	1195		9.93 ± 0.03^a^	2.03 ± 0.02^b^	a, b
25	cis‐Dihydrocarveol	7.566	1030	7.63 ± 0.03^a^			a, b
26	cis‐Isopiperitenol	7.599	970			1.04 ± 0.02^a^	a, b
27	Verbenol	7.727	1085			0.47 ± 0.01^a^	a, b
28	*trans*‐carveol	7.813	1217	1.24 ± 0.02^c^	9.14 ± 0.03^a^	5.81 ± 0.03^b^	a, b
29	*trans*‐Carvyl acetate	7.941	1016	7.62 ± 0.03^a^	5.65 ± 0.03^b^	4.33 ± 0.03^c^	a, b
30	Carveol	8.021	1160		5.56 ± 0.03^a^	4.33 ± 0.03^b^	a, b
31	d‐Carvone	8.043	1242	4.25 ± 0.03^b^	8.46 ± 0.03^a^	4.1 ± 0.02^c^	a, b
32	Perillol	8.481	1271	3.85 ± 0.01^a^			a, b
33	Carvone oxide	8.529	980	1.52 ± 0.01^a^			a, b
34	β‐Terpenyl acetate	8.556	1190			2.32 ± 0.03^a^	a, b
35	Neoisomenthol acetate	9.177	1007			0.81 ± 0.01^a^	a, b
36	Levomenol	11.09	1060		2.08 ± 0.02^a^		a, b
Sesquiterpene hydrocarbons							
37	Ligustral	6.85	1394	0.42 ± 0.01^b^		1.53 ± 0.02^a^	a, b
38	delta‐Elemene	7.631	1339			0.65 ± 0.01^a^	a, b
39	γ‐cadinene	8.604	1477			1.6 ± 0.02^a^	a, b
40	α‐himachalene	9.246	1447			3.42 ± 0.03^a^	a, b
41	Copaene	9.353	1376	3.90 ± 0.02^a^	1.85 ± 0.02^b^		a, b
42	Caryophyllene	9.669	1418		1.76 ± 0.02^a^	0.22 ± 0.01^b^	a, b
43	*trans*‐ alpha‐Bergamotene	9.706	1105		1.49 ± 0.01^a^		a, b
44	Aromadendrene	9.824	1439			0.38 ± 0.01^a^	a, b
45	α‐cis‐Bergamotene	10.29	1473		2.93 ± 0.02^a^		a, b
46	β‐bisabolene	11.1	1509	1.25 ± 0.02^a^		1.28 ± 0.02^a^	a, b
47	gamma‐Muurolene	11.51	1477	0.43 ± 0.01^b^	8.06 ± 0.03^a^		a, b
Oxygenated sesquiterpenes							
48	Nerolidol	9.177	1564			0.81 ± 0.01^a^	a, b
49	*trans*‐crysanthaol	9.904	1670			1.29 ± 0.02^a^	a, b
50	Caryophyllene oxide	11.11	1581	1.53 ± 0.02^b^	3.93 ± 0.01^a^	1.25 ± 0.02^c^	a, b
51	tau‐Cadinol	11.51	1501		2.72 ± 0.03^a^		a, b
Other hydrocarbons							
52	Propellane	7.187	1005			0.73 ± 0.01_a_	a, b
53	*trans*‐Decalin	7.566	1045	15.26 ± 0.03^a^			a, b
54	Isophorone	8.331	1015	1.53 ± 0.01^a^			a, b
55	Perillartine	8.374	1099	2.36 ± 0.02^a^			a, b
56	Fulvene	8.84	1290			1.67 ± 0.02^a^	a, b
57	Santolina triene	8.87	908			1.65 ± 0.02^a^	a, b
58	Octillion	9.102	978	7.72 ± 0.02^a^		1.38 ± 0.01^b^	a, b

*Note:* Values represent the mean ± standard deviations calculated from three independent trials. Different letters in the superscripts express significant variances in the EOs extracted from *C. sinensis* peels using various extraction methods. “A” illustrates retention time, while “B” signifies retention index. Retention index‐based identification is represented by “a” while “b” exhibits comparison of mass spectra‐based identification.

Similarly, a total of twenty‐five oxygenated monoterpenes HCs were found, including eucalyptol, linalool oxide, linalool, limonene oxide, citronellal, camphor, *trans*‐isopiperitenol, alpha‐campholenal, carvomenthone, carvomenthenol, citral, geraniol, estragole, cis‐dihydrocarveol, cis‐isopiperitenol, verbenol, *trans*‐carveol, *trans*‐carvyl acetate, carveol, d‐carvone, perillol, carvone oxide, β‐terpenyl acetate, neoisomenthol‐acetate, and levomenol. Among all these components, estragole and *trans*‐carveol appeared as primary oxygenated monoterpene HCs with a concentration range of 2.03% to 9.93% and 1.24% to 9.14%, respectively. While the other oxygenated monoterpene HCs were identified in relatively smaller concentrations, as mentioned in Table 3. Correspondingly, various volatile contents of sesquiterpenes HCs, oxygenated sesquiterpenes HCs, and other HCs were also detected. A study reported that d‐limonene is an essential component of citrus oils, recognized for its pleasant lemon‐like aroma. It is widely used as a flavoring and aroma addition in cosmetics, meals, and industrial solvents (Kim et al. 2013). It was discovered that d‐limonene can help protect against chronic and degenerative health conditions. The beneficial effect has been extensively reported, demonstrating its potential as a valuable preventive agent (Anandakumar et al. 2021). Aligning with the literature reports, the EO extracted from orange peels yielded a composition rich in d‐limonene, which constitutes 90 to 98% of the oil while making up 3.8 to 5.3% of the orange peel by weight (Siddiqui et al. 2022). It has been revealed that across twelve cultivars of *C. sinensis* , d‐limonene was the major compound (73.9% to 97%), followed by trace amounts of nerol, geraniol, and linalool (Geraci et al. 2017). Furthermore, *C. sinensis Osbeck* EO was found to be dominated by monoterpenes and sesquiterpene compounds, comprising 96.03%. Limonene was a primary compound (77.49%), followed by myrcene (6.27%), α‐farnesene (3.64%), γ‐terpinene (3.34%), α‐pinene (1.49%), sabinene (1.29%), and other minor compounds (Tao et al. 2009). Previous research indicates that the main component of cold‐pressed *C. sinensis* Osbeck cv. Newhall EO and light‐phase EO was limonene, accounting for 85.32% and 60.44%, respectively (Guo et al. 2018). Moreover, *C. sinensis* L. Osbeck EOs from Uganda and Rwanda were reported to have limonene as the primary compound (87.9% and 92.5%), together with a small amount of myrcene, α‐pinene, linalool, octanal, and decanal (Njoroge et al. 2009).

A previous study has revealed that compounds with less volatility release at high temperatures, while highly volatile compounds emerge at low temperatures (Naz et al. 2023). It was also suggested that volatile contents and their concentrations depend upon the genotype, tapping methods, plant part being used, extraction method, extraction time, and plant maturity (Ayub, Ijaz, et al. 2024). Moreover, extended exposure to high temperatures can lead to the formation of by‐products by degradation or solubilization of the pure compounds, which ultimately decreases both the EO yield and selectivity (Raza et al. 2024).

### 3.3 Anti‐Microbial Activity

The antibacterial activity of *C. sinensis* EO extracted by different extraction techniques was estimated against a Gram‐positive bacterium ( *S. aureus* ) and a Gram‐negative bacterium ( *E. coli* ). To determine the diameter of the inhibition zone, the agar well diffusion assay was used for both bacterial and fungal strains. The resazurin microtitre plate assay and microdilution broth assay were used to measure the MIC values for bacterial and fungal strains, respectively. The higher the Inhibition zone and the lower the MIC value, the greater the antibacterial activity of the EO.

The inhibition zone values against the *E. coli* strain ranged between 12.12 and 19.10 mm. While against *S. aureus* , values ranged between 18.46 and 24.09 mm. The MIC values against both tested strains were 12.50 to 50 and 3.12 to 25.00 μg/mL, respectively. For comparison, the positive control (ampicillin) illustrated stronger activity. The highest inhibition zone, 24.09 mm, and the lowest MIC, 3.12 μg/mL, were observed against the *S. aureus* strain, indicating that this strain is more susceptible. The maximum antibacterial potential was revealed by SHS‐extracted EO, indicating that it is more effective than all the other extracted EOs. Results are declared in Table 4.

**TABLE 4:** Antimicrobial activity of *C. sinensis* EOs extracted by various techniques.

Antibacterial activity of EOs extracted by different extraction methods				
Microorganisms	HD	SD	SHSE	Ampicillin
Inhibition zone (mm)				
*Escherichia coli*	12.12 ± 0.03^d^	17.02 ± 0.11^c^	19.10 ± 0.09^b^	30.72 ± 0.02^a^
*Staphylococcus aureus*	18.46 ± 0.01^d^	21.97 ± 0.12^c^	24.09 ± 0.15^b^	31.36 ± 0.09^a^
Minimum inhibitory concentration (μg/mL)				
*Escherichia coli*	50 ± 0.58^a^	25 ± 1.52^b^	12.50 ± 1.32^c^	0.78 ± 0.00^d^
*Staphylococcus aureus*	25.00 ± 0.09^a^	6.25 ± 0.15^b^	3.12 ± 0.04^c^	0.78 ± 0.01^d^

Antifungal activity of EOs extracted by different extraction methods				
Microorganisms	HD	SD	SHSE	Fluconazole
Inhibition zone (mm)				
*Fusarium solani*	15.90 ± 0.04^d^	19.11 ± 0.02^c^	20.83 ± 0.05^b^	24.78 ± 0.03^a^
*Aspergills niger*	10.87 ± 0.01^d^	15.64 ± 0.03^c^	17.56 ± 0.04^b^	22.87 ± 0.03^a^
Minimum inhibitory concentration (μg/mL)				
*Fusarium solani*	25.00 ± 0.75^a^	6.25 ± 0.18^b^	3.12 ± 0.10^c^	1.56 ± 0.05^d^
*Aspergillus niger*	50.00 ± 2.15^a^	25.00 ± 1.05^b^	6.25 ± 0.25^c^	3.12 ± 0.16^d^

*Note:* Values express the mean ± standard deviations of three independent trials. Different letters in the superscripts denote significant variances in the EOs extracted from *C. sinensis* peels using various extraction methods.

Antifungal potential of the extracted EOs was measured against *F. solani*, and *A. niger* fungal strains. Findings are presented in Table 4. The zone of inhibition ranged from 15.90 to 20.83 mm against *F. solani* strain, while 10.87 to 17.56 mm against *A. niger* . On the other hand, MIC values of both tested strains ranged from 3.12 to 25.00 μg/mL and from 6.25 to 50.00 μg/mL, respectively. For comparison, the control, fluconazole, revealed greater antifungal potential (Table 4). Overall, *F. solani* was found to be more susceptible with a greater inhibition zone (15.90 to 20.83 mm) and lower MIC value (3.12 to 25.00 μg/mL), and SHS extracted EO appeared as more active among all the extracted EOs. The diffusion rate of EO components into the agar determines the extent of the inhibitory zone in agar disc diffusion (Kalemba and Kunicka 2003). The ability of EOs to blend into the lipids of the bacterial cell membrane disrupts the cell structure and increases the membrane permeability. This ultimately causes the bacterial cells to leak large amounts of vital chemicals and ions, and as a result, leads to cell death (Oulkheir et al. 2017).

The findings demonstrate that the employed extraction processes significantly impact the antibacterial properties of *C. sinensis* EOs. The difference in antibacterial activity measurements between various extraction methods may be attributed to slight variations in the chemical compounds of the EOs (Glišić et al. 2007). Some extraction procedures, due to their conditions, may lead to the loss of components that contribute to the antibacterial activity of the EOs (Reyes‐Jurado et al. 2015).

(Hacib et al. 2024) reported that *C. sinensis* EOs extracted by hydrodistillation and microwave‐assisted extraction exhibited strong antibacterial activity against *Escherichia coli* , *Listeria monocytogenes* , and *Agrobacterium* spp., with inhibition zone diameters ranging from 70 to 84 mm under disc diffusion conditions. Additionally, it was found that the antibacterial potential of *C. sinensis* and *C. reticulata* peel EOs was similar against both *E. coli* and *B. subtilis* bacterial strains, although *S. aureus* bacteria revealed decreased resistance toward *C. sinensis* peel EO (Zhang et al. 2022). Literature studies showed that the EOs extracted from lemon and orange peels exhibited the most potent antibacterial action against *Staphylococcus aureus* (Akarca and Sevik 2021). It was previously declared that EOs extracted from grapefruit, orange, lemon, and mandarin exhibited antifungal potential, where orange EO was most effective against *A. niger* , mandarin EO demonstrated maximum effectiveness against *A. flavus* , and grapefruit EO produced the strongest inhibition impact on *P. chrysogenum* and *P. verrucosum* growth (Viuda‐Martos et al. 2008).

The antifungal effectiveness of *C. sinensis* EO reaches 100% against *Fusarium culmorum* and 86.66% against *Aspergillus flavus* fungal growth at 50 μL/mL dose levels, and these results improve when combined with *Eucalyptus camaldulensis* EO, which exhibits eco‐friendly fungicide potential (Elgat et al. 2020). Previous studies indicate that the significant antibacterial potential of *C. sinensis* EO is due to the presence of limonene, which can damage microbes' cell membranes (Gupta et al. 2021). It was exposed that the antimicrobial effect of *C. sinensis* Osbeck might be due to the synergistic effect of limonene, linalool, α‐pinene, β‐pinene, and some other compounds (Tao et al. 2009). The findings demonstrate that *C. sinensis* EOs possess significant antimicrobial potential, which is likely influenced by their chemical composition.

### 3.4 Antioxidant Activity

The antioxidant activity of *C. sinensis* EO was assessed using hydrogen peroxide, FRAP, and DPPH assays. Table 5 presents the findings of antioxidant activities. 2,2‐Diphenyl‐1‐picrylhydrazyl (DPPH) is a stable free radical commonly used to evaluate the radical‐scavenging activity of plant extracts. The method depends on DPPH reduction through which essential oils (EO) donate a hydrogen atom to convert DPPH into DPPHH and change the solution color from purple to colorless (Ayub, Ijaz, et al. 2024).

**TABLE 5:** Antioxidant potential of the *C. sinensis* EOs extracted by different extraction methods.

Extraction methods	% DPPH	% Hydrogen peroxide	Ferric reducing antioxidant power (mg/mL)
Hydro distillation	63.61 ± 0.25^d^	27.09 ± 0.35^d^	77.08 ± 1.21^c^
Steam distillation	64.50 ± 0.33^c^	34.06 ± 0.33^c^	97.07 ± 2.01^b^
Superheated steam extraction	67.66 ± 0.16^b^	51.1 ± 0.19^b^	214.19 ± 2.24^a^
Gallic acid	82.41 ± 0.18^a^	—	
Ascorbic acid	—	58.28 ± 0.42^a^	

*Note:* Values express the mean ± standard deviations of three independent trials. Different letters in the superscripts denote significant variances in the EOs extracted from *C. sinensis* peels using various extraction methods.

The results showed that SHS extracted EO possessed the maximum antioxidant potential, 67.66%, as determined by the DPPH assay. Meanwhile, hydro‐distilled EO exhibited the lowest antioxidant potential of 63.61% against the DPPH assay. In comparison, the standard gallic acid exhibited greater activity (Table 5).

The hydrogen peroxide assay evaluates antioxidant activity by observing the rise in absorbance brought on by H_2_O_2_ oxidation (Özkan and Erdoğan 2011). The results showed that the maximum value of H_2_O_2_ scavenging activity (51.1%) was found in the SHS extracted EO, while the minimum potential (27.09%) was found in the hydro‐distilled EO. In comparison, the standard ascorbic acid exhibited greater activity (Table 5). Another frequently employed technique to evaluate a compound's antioxidant activity is the FRAP assay. In a redox‐linked reaction, it depends on the antioxidants' capacity to function as reducing agents. In the FRAP assay, ferric ions (Fe^3+^) are reduced to ferrous ions (Fe^2+^) in an acidic environment through a colorimetric process. As a result of this reduction, a colored complex is formed, and its intensity is directly correlated with the sample's overall antioxidant capacity. The intensity of this color change is measured using a spectrophotometer at a specific wavelength (Gülçin et al. 2011).

Fluctuations in the antioxidant activity of *C. sinensis* EOs were noted across several extraction regimens due to differences in the extraction technique used. Overall, the total antioxidant contents ranged between 77.08 and 214.19 mg/mL. The EO isolated by SHSE exhibited the maximum antioxidant contents (214.19 mg/mL), while the minimum antioxidant contents, 77.08 mg/mL, were found in *C. sinensis* EO extracted through the HD method.

Previously, *C. sinensis* EO was reported to have 6 to 23% antioxidant potential against the DPPH assay (Toscano‐Garibay et al. 2017). Moreover, it was declared that *C. sinensis* EO demonstrated antioxidant potential as a DPPH free radical scavenger, with an IC_50_ value of 9.45 μL/mL (Singh et al. 2010). It has been published that *C. sinensis* EO revealed an IC_50_ value of 752.26 μg/mL, with 55.56% β‐carotene/linoleic acid inhibition of 55.56%, while having total phenolic contents of 10.53 μg/mg (Ben Miri et al. 2018). Some earlier research found that monoterpenes such as limonene, α‐terpinene, terpinolene, γ‐terpinene, geraniol, myrcene, α‐terpineol, linalool, and β‐pinene in citrus plants give them strong antioxidant properties (Manzur et al. 2023). It was also found that citrus EOs have antioxidant properties because they are rich in phenolics (Kamal et al. 2013). Another study suggested that the antioxidant effectiveness of citrus EO might be due to the combined effect of γ‐terpinene and d‐limonene compounds (Li et al. 2022).

Overall, the findings concluded that EO extracted by SHSE exhibited the highest antioxidant potential in all the selected assays (DPPH, H_2_O_2_, and total antioxidant content by FRAP), which may be attributed to the maximum concentration of volatile compounds.

### 3.5 Molecular Docking

Essential oils are considered biologically effective due to the synergistic effect of their volatile components. To verify whether each component was bioactive or not, a total of fifteen compounds were selected to evaluate their interactions with six selected receptors (1CEX, 1KZN, 2CDU, 2I80, 3NRZ, and 7BLY). While BHT was examined for comparison. Results are demonstrated in Table 6. Each compound revealed a specific energy value with each protein's binding site compared to the others. Although d‐limonene declared comparable binding energies to other selected compounds, it was selected for further analysis along with three most favorable compounds, trans‐carvyl acetate, β‐terpinyl acetate, and α‐himachalene, because it appeared as a major compound in GC–MS analysis. The binding scores of d‐limonene with the selected receptors were in the following decreasing order: 3NRZ < 2CDU < 1KZN = 7BLY < 2I80 < 1CEX. Similarly, *trans*‐carvyl acetate revealed the decreasing order, 3NRZ < 2CDU < 7BLY < 2I80 = 1KZN < 1CEX. The compound, β‐terpinyl acetate, declared binding score in decreasing order, 2CDU < 1KZN = 2I80 < 3NRZ < 7BLY = 1CEX, while the reducing order of α‐himachalene was 2I80 < 2CDU < 3NRZ < 1KZN < 7BLY < 1CEX. The findings exhibited that each bioactive ligand revealed a maximum binding score with the 1CEX receptor, which showed the least protein–ligand binding interactions.

**TABLE 6:** Energy (kcal/mol) values of ligand–protein interactions between the selected ligands and proteins.

Compounds	1CEX	1KZN	2CDU	2I80	3NRZ	7BLY
β‐pinene	−4.5	−4.7	−6.1	−6.0	−5.5	−4.8
d‐limonene	−4.9	−5.8	−6.0	−5.4	−6.3	−5.8
β‐phellandrene	−4.8	−5.7	−6.3	−6.6	−6.4	−5.6
l‐limonene	−4.9	−5.8	−6.0	−6.1	−6.1	−5.8
Camphene	−4.6	−4.5	−6.1	−5.7	−5.7	−4.6
Linalool oxide	−4.9	−4.9	−5.7	−5.9	−5.9	−5.1
Linalool	−4.2	−5.1	−5.7	−6.1	−5.5	−4.8
p‐menthane	−4.3	−5.5	−6.1	−6.5	−5.5	−5.3
trans‐carvyl acetate	−5.1	−6.1	−6.5	−6.1	−6.9	−6.3
d‐carvone	−5.0	−6.0	−6.1	−5.8	−6.5	−6.2
Estragole	−4.7	−5.6	−5.8	−6.3	−6.2	−5.1
Carveol	−4.9	−6.0	−6.2	−6.3	−6.3	−6.5
trans‐carveol	−5.0	−5.8	−5.9	−5.6	−6.7	−6.3
β‐Terpenyl acetate	−5.8	−6.2	−6.8	−6.2	−6.0	−5.8
α‐himachalene	−5.4	−6.2	−7.4	−7.6	−6.9	−6.0
BHT	−5.5	−5.7	−4.9	−5.3	−7	−7.3

**FIGURE 1:**
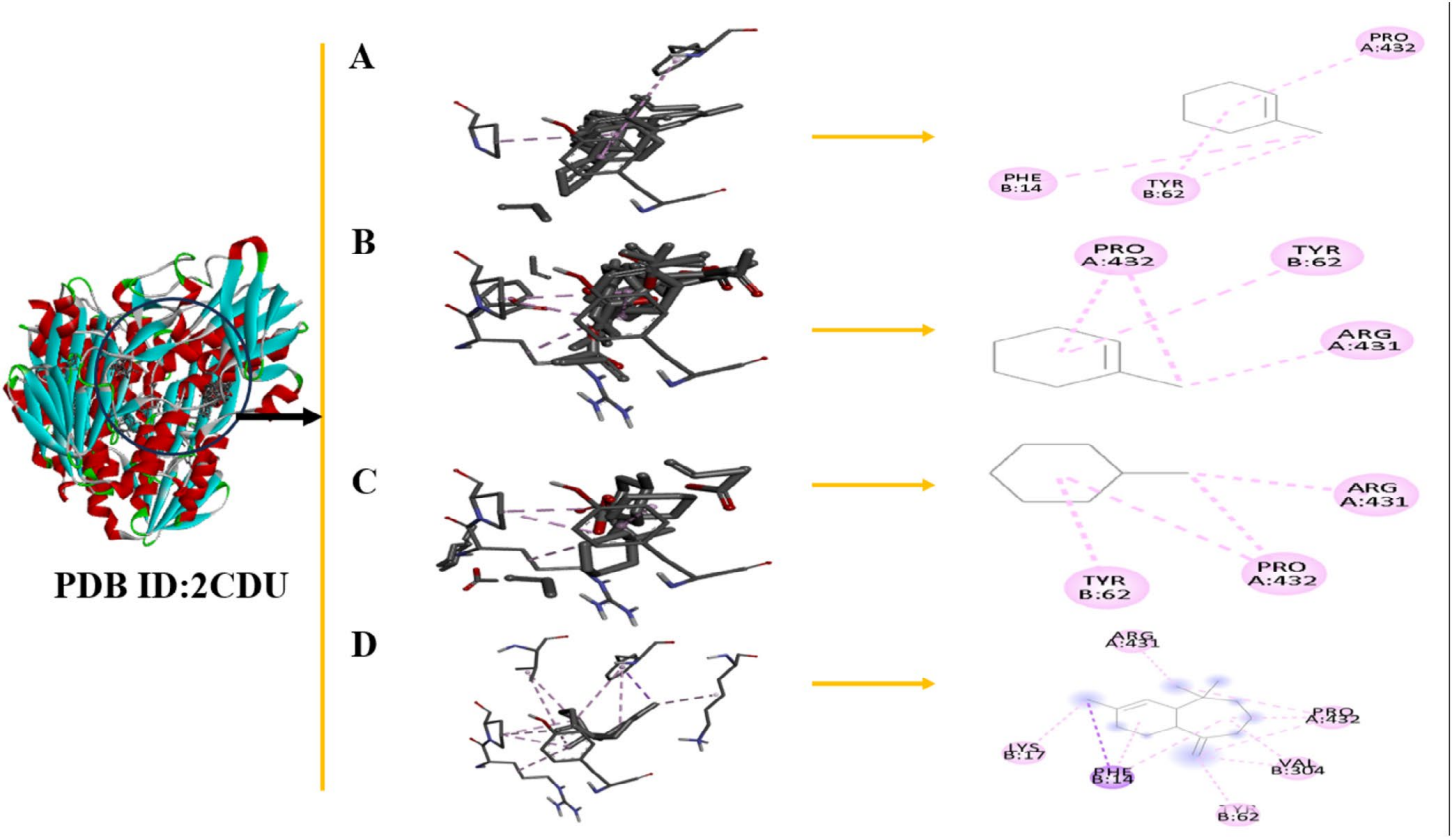
Identification of active site locations by ligand–protein interactions of PDB ID: 2CDU and (A) D‐limonene, (B) *trans*‐carvyl acetate, (C) β‐terpinyl acetate, and (D) α‐himachalane.

**FIGURE 2:**
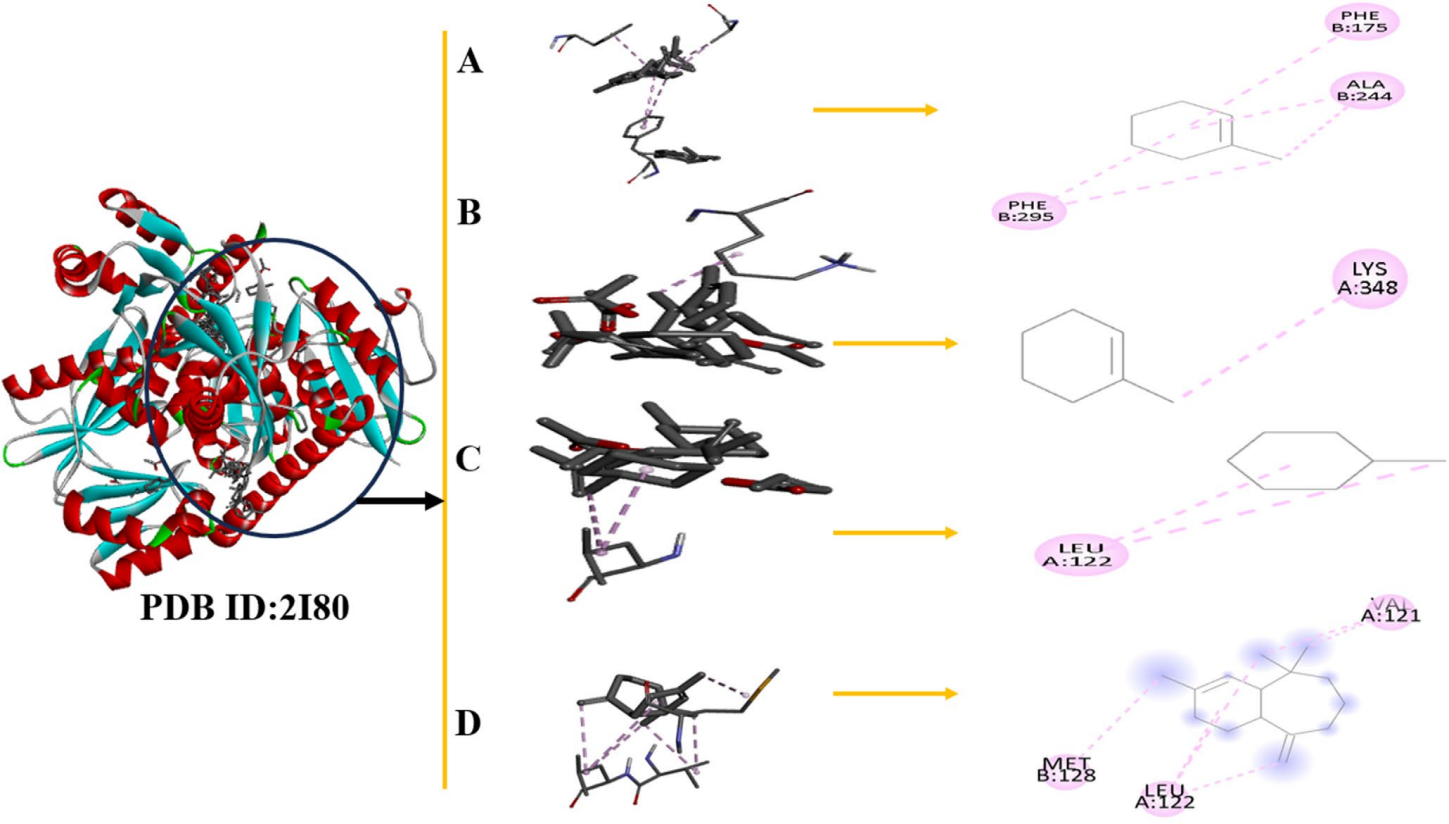
Identification of active site locations by ligand–protein interactions of PDB ID: 2I80 and (A) D‐limonene, (B) *trans*‐carvyl acetate, (C) β‐terpinyl acetate, and (D) α‐himachalane.

**FIGURE 3:**
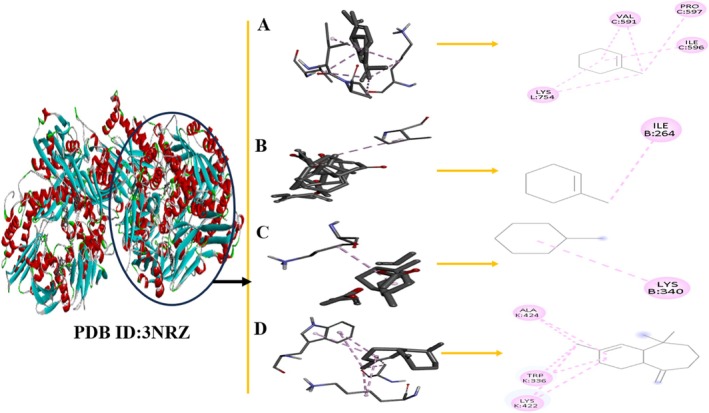
Identification of active site locations by ligand–protein interactions of PDB ID: 3NRZ and (A) *d*‐limonene, (B) *trans*‐carvyl acetate, (C) β‐terpinyl acetate, and (D) α‐himachalane.

The investigation of the 2CDU protein structure revealed that the residues PHE14, PRO432, ARG431, TYR62, and VAL304, located in the active site, exhibited sufficient ligand–protein interactions. The active site of the 2CDU protein is shown to have many residues interacting with it, as shown in Figure 1. These interactions are essential in stabilizing the binding site and promoting the affinity between the ligand and protein structures. The elaborate description of these residues gives valuable information regarding the molecular mechanism of ligand recognition and binding efficiency. According to the obtained binding energies, PHE175, PHE295, ALA244, LYS348, LEU122, and MET128 were identified as effective residues in the 2I80 protein structure, as shown in Figure 2. The intense analysis of the 3NRZ structure highlighted the essential participation of VAL591, PRO597, ILE596, LYS754, ILE264, LYS340, ALA424, TRP336, and LYS422 residues in the active region, indicating their crucial contribution to promoting ligand interactions and maintaining the structure of the molecular assembly. The presence of these residues was carefully studied, and their contribution was graphically illustrated in Figure 3, highlighting their role in binding efficacy and structural integrity.

Figures 1, 2, 3 depict that among all the ligands, d‐limonene, *trans*‐carvyl acetate, β‐terpinyl acetate, and α‐himachalene revealed strong interaction. Additionally, Figures 1, 2, 3 emphasize that 2CDU, 2I80, and 3NRZ kept stable interactions with the selected ligands.

**FIGURE 4:**
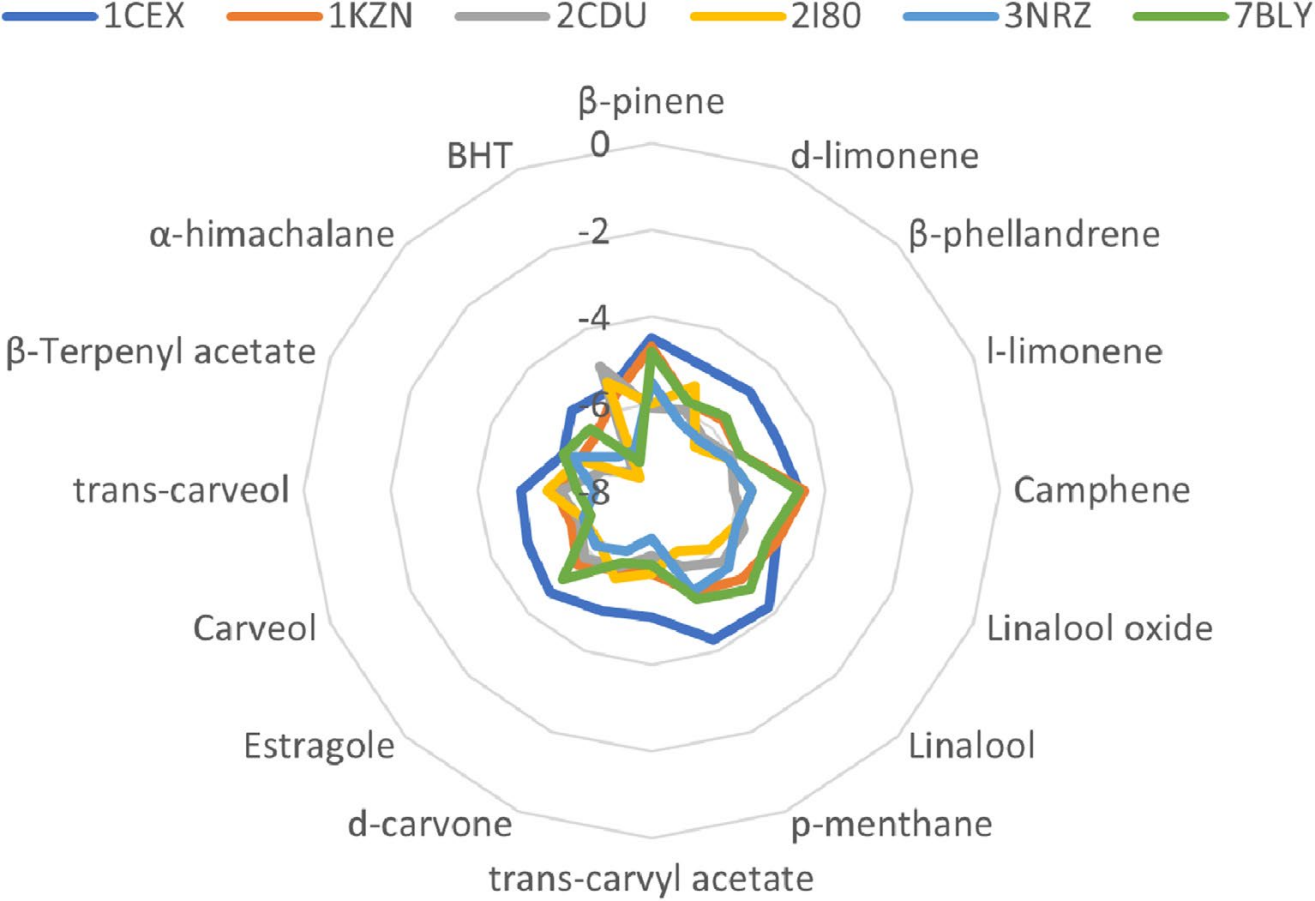
Comparison of binding energy and effectiveness between the selected ligands and proteins.

Extensive analysis in Figure 4 concluded that the most selected proteins, 2CDU, 2I80, and 3NRZ, have a significant interaction with the extracted compounds from *C. sinensis* EO. Furthermore, it seems that the proteins 1CEX, 1KZN, and 7BLY did establish comparable contacts between crucial residues and ligands in their active sites.

### 3.6 Density Functional Theory

#### 3.6.1 Optimized Structures

**FIGURE 5:**
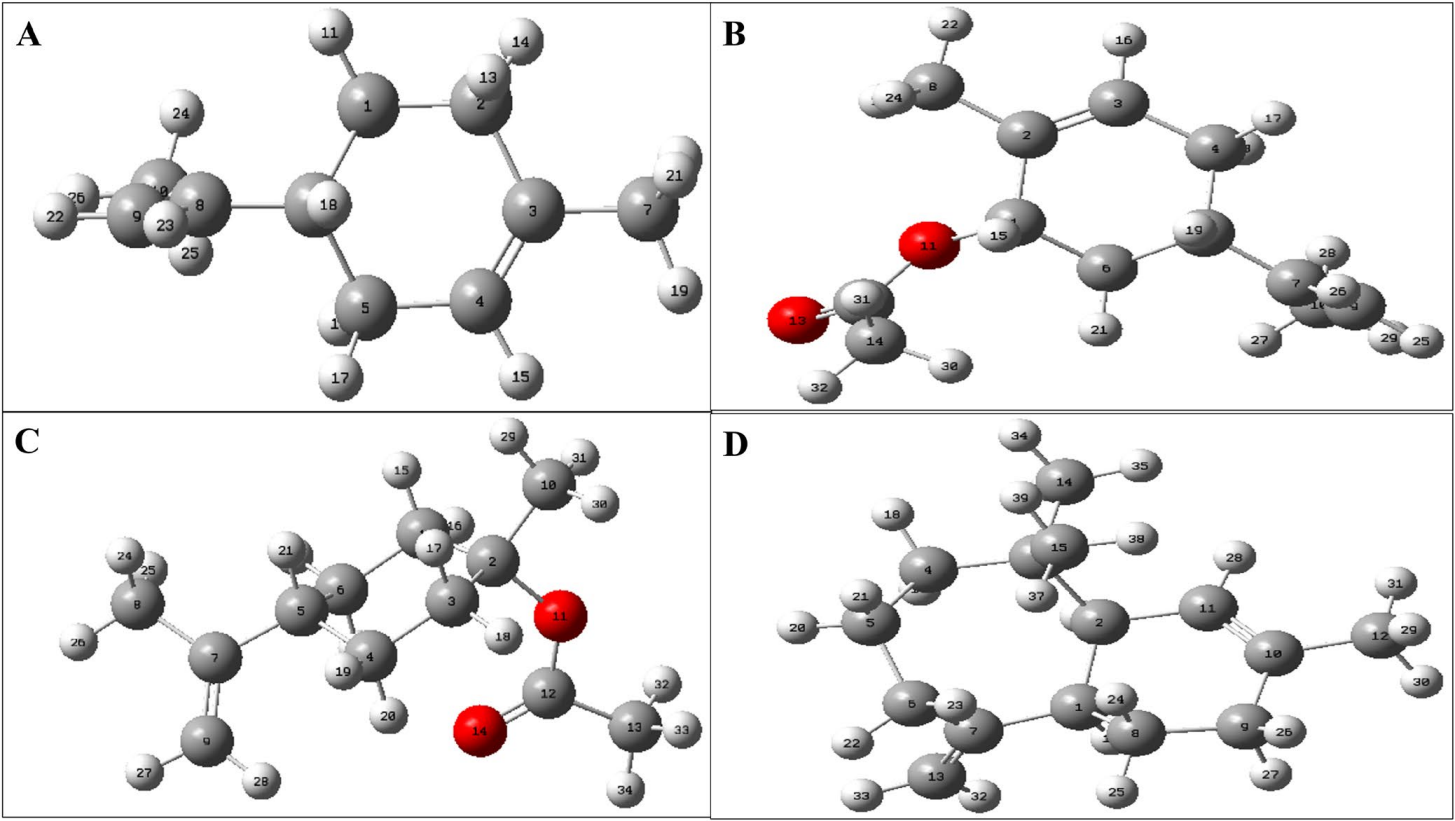
Optimized geometries of (A) *d*‐limonene, (B) *trans*‐carvyl acetate, (C) β‐terpinyl acetate, and (D) α‐himachalane.

Density functional theory at the B3LYP/6–31 G (d,p) level was used to optimize the molecules d‐limonene, *trans*‐carvyl acetate, β‐terpinyl acetate, and α‐himachalene. Figure 5 shows the optimized structures of the compounds.

#### 3.6.2 Frontier Molecular Orbitals

A key factor in determining molecular reactivity is the frontier molecular orbitals, which consist of the lowest unoccupied molecular orbital (LUMO) and the highest occupied molecular orbital (HOMO). The gap or difference between HOMO and LUMO energy levels is a significant factor in measuring antioxidant activity. Quantum chemical descriptors based on the HOMO and LUMO energies affect the reactivity of antioxidants. They are essential for understanding the charge transfer interactions that exist between reactive oxygen species (ROS) and antioxidants (Mahmoudi et al. 2021).

A stable molecule exhibits a high energy gap and low reactivity, while a molecule with a small energy gap proves to be highly reactive due to its lack of stability. Molecular reactivity is key in determining whether a substance possesses antioxidant properties. High reactivity enables a molecule to neutralize more free radicals, making it a more effective antioxidant. Moreover, if the molecule has low reactivity, it has little ability to act as an oxidative agent (Anjam et al. 2025). It is essential to understand this relationship to evaluate the effectiveness of antioxidants in both chemical and biological systems.

**FIGURE 6:**
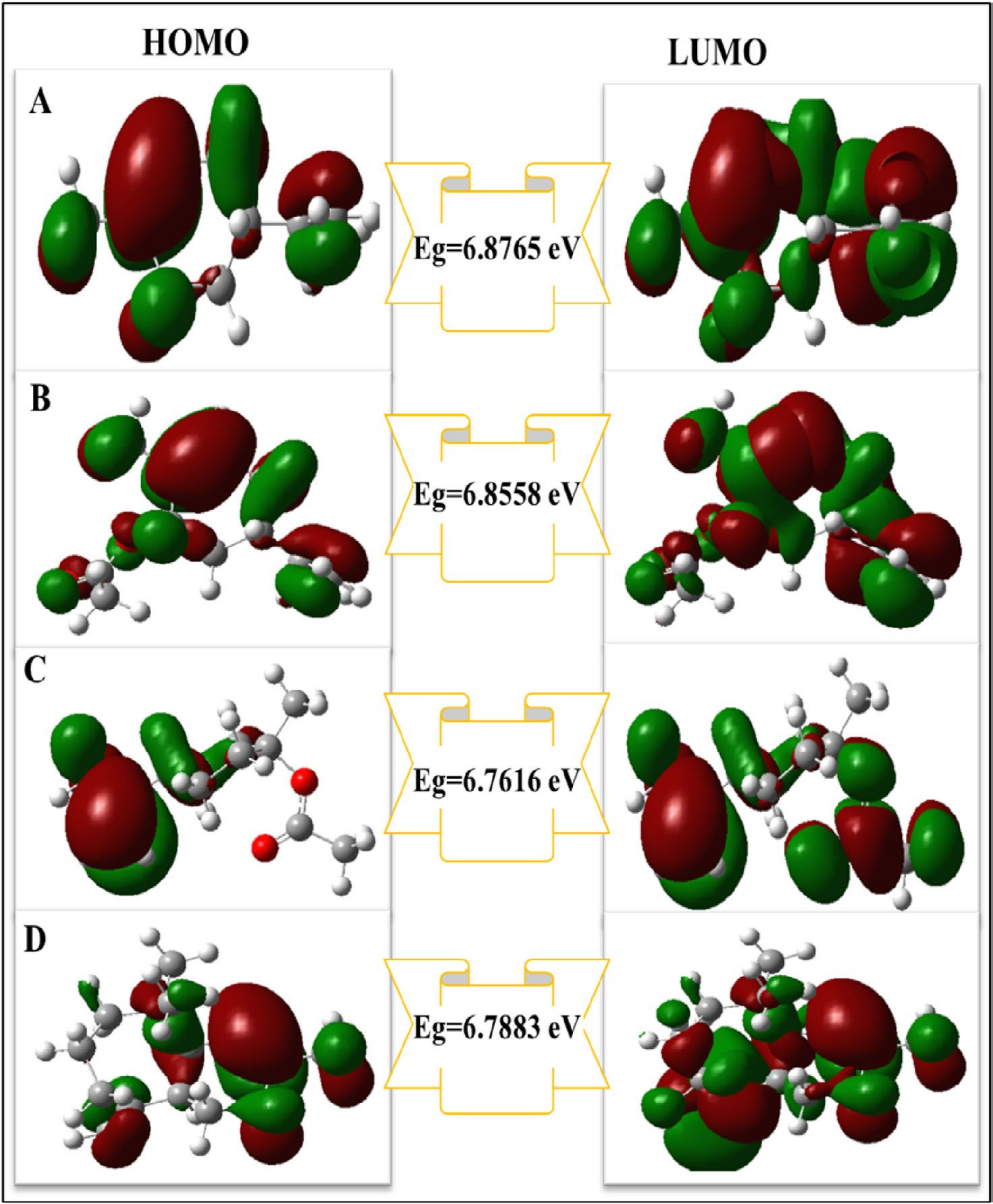
Electron density lobes of HOMO and LUMO levels of energy for (A) *d*‐limonene, (B) *trans*‐carvyl acetate, (C) β‐terpinyl acetate, and (D) α‐himachalane.

Overall, four compounds, including d‐limonene, *trans*‐carvyl acetate, β‐terpinyl acetate, and α‐himachalene, were selected to find out the best antioxidant compound through DFT studies at a level of B3LYP/6–31 G (d,p). Findings are expressed in Table 7. The HOMO values for all the compounds were −6.1359 (eV), −6.6044 (eV), −6.3437 (eV), and −6.1756 eV, respectively. On the other hand, the values of the LUMO level are 0.7406 (eV), 0.2514 (eV), 0.4179 (eV), and 0.6127 eV, respectively. As compared to others, β‐terpinyl acetate exhibited the lowest energy gap value, 6.7616 eV, indicating that this molecule has more antioxidant potential compared to the other three compounds: d‐limonene, *trans*‐carvyl acetate, and α‐himachalane. The HOMO and LUMO lobes are shown in Figure 6.

**TABLE 7:** Comparison of HOMO, LUMO energy values, and energy gap of the four examined molecules.

Molecules	HOMO (eV)	LUMO (eV)	Energy gap (eV)
d‐limonene	−6.1359	0.7406	6.8765
*trans*‐carvyl acetate	−6.6044	0.2514	6.8558
β‐terpinyl acetate	−6.3437	0.4179	6.7616
α‐himachalene	−6.1756	0.6127	6.7883

β‐terpinyl acetate features a cyclohexane ring, which facilitates a more efficient electronic transition. Moreover, this molecule has an acetate group, which is composed of two electronegative oxygen atoms. These oxygen atoms withdraw electrons from other atoms, causing more electronic transitions.

#### 3.6.3 Global Reactivity Parameters

Ionization potential (IP), electron affinity (EA), chemical softness, and chemical hardness are collectively known as global reactivity parameters of a molecule. The energy levels, HOMO and LUMO, help to evaluate the global reactivity parameters. The IP and EA are used to find the chemical softness and hardness of a molecule. It has been established that a higher value of softness indicates a highly reactive molecule, while a lower value is associated with being less reactive. On the other hand, the greater value of chemical hardness shows less reactivity of the molecule, and vice versa (Zochedh et al. 2022). The calculated values are presented in Table 8.

**TABLE 8:** Ionization potential, electron affinity, softness, and hardness parameters of all the selected molecules.

Molecules	Ionization potential (eV)	Electron affinity (eV)	Softness (eV^−1^)	Hardness (eV)
d‐limonene	6.1359	−0.7406	0.1454	3.4383
trans‐carvyl acetate	6.6044	−0.2514	0.1458	3.4279
β‐terpinyl acetate	6.3437	−0.4179	0.1478	3.3808
α‐himachalane	6.1756	−0.6127	0.1473	3.3942

The softness values of the selected molecules, d‐limonene, *trans*‐carvyl acetate, β‐terpinyl acetate, and α‐himachalene, were 0.1454, 0.1458, 0.1478, and 0.1473, respectively. While all the molecules exhibited hardness values of 3.4383, 3.4279, 3.3808, and 3.3942, respectively. Overall, it was observed that the compound β‐terpinyl acetate exhibited the highest value of softness (0.1478) and the lowest hardness (3.3808), indicating that it is the most effective antioxidant agent among the selected molecules, as demonstrated in Table 8.

#### 3.6.4 Molecular Electrostatic Potential

**FIGURE 7:**
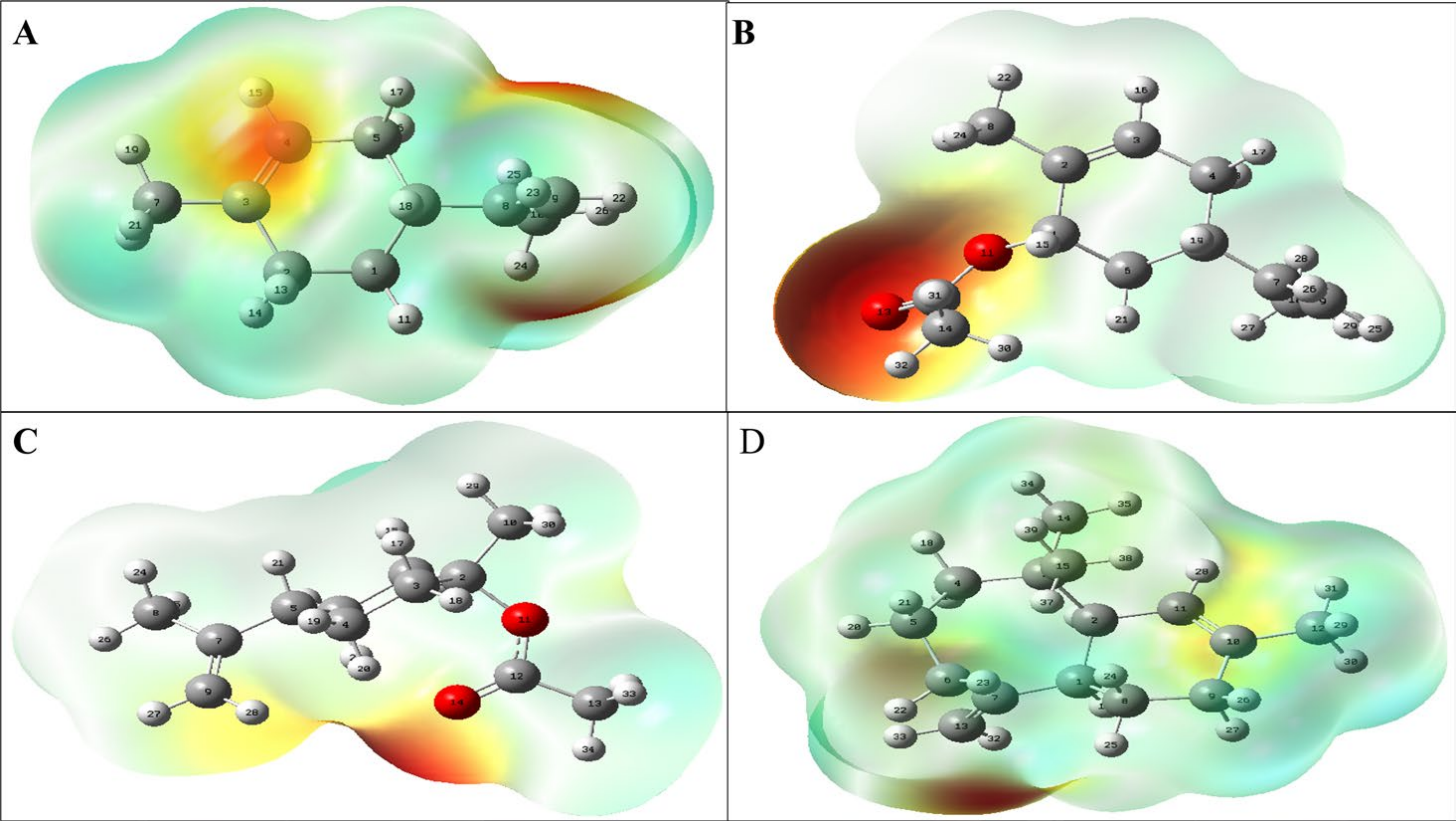
Structures showing MEP analysis of (A) *d*‐limonene, (B) *trans*‐carvyl acetate, (C) β‐terpinyl acetate, and (D) α‐himachalane.

The MEP analysis reveals the distribution of charges inside a molecule in three dimensions. Moreover, this analysis highlights the different sites on the molecule, including electron‐rich, electron‐deficient, and neutral sites, by representing specific colors. The MEP analysis of the selected compounds was conducted at the B3LYP/6–31 G (d,p) level of the DFT. A specific color provides significant information about the corresponding region. Red color indicates the electron‐rich part, blue color illustrates a part with electron deficiency, and the green color illustrates the neutral part of the compound (Zochedh et al. 2022). The MEP diagrams for the selected molecules are displayed in Figure 7.

The molecule (A), d‐limonene, is represented by the red color at atom number 4, indicating that this region is electron‐rich and a favorable site for electrophilic attack. *Trans*‐carvyl acetate, molecule (B), showed red color at atom numbers 11, 13, 14, 31, and 32, representing the electron‐rich part. The atom number 14 of compound (C), β‐terpinyl acetate, exhibits a red coloration, which signifies a region of strongly negative electrostatic potential and a nucleophilic center. Despite being an oxygen atom as well, atom 11 lacks red color, which suggests a reduced electron density there, due to its bonding environment. Moreover, in the mep structure of α‐himachalene, compound (D), charge delocalization is indicated by the brownish zones around atoms 6 and 13, which indicate a mildly electron‐rich region.

## 4 Conclusion

Essential oils are widely used for many medicinal purposes and as fragrance agents in various industries. The present work investigated the *C. sinensis* EO extraction through conventional methods (HD and SD) as well as an emerging SHSE technique. The GC–MS analysis identified a diverse profile of chemical components and found d‐limonene as a major compound in the extracted EOs. The highest EO yield and superior antimicrobial and antioxidant activities were observed in the EO extracted by SHSE, suggesting that this approach is more beneficial to extract a high quantity of volatile bioactive components. The biological activities of the extracted EOs were attributed to the synergistic effect of their minor and major compounds. Molecular docking supported this, as all selected compounds showed efficient binding to the target proteins. Moreover, d‐limonene showed comparable binding energy, while *trans*‐carvyl acetate, β‐terpinyl acetate, and α‐himachalene exhibited lower binding scores and showed high interactions with receptors. While DFT analysis provided deeper molecular‐level insights into the reactivity and stability of key compounds, supporting their therapeutic potential. DFT justified β‐terpinyl acetate as the most active antioxidant compound, attributed to its lowest energy gap, maximum chemical softness, and minimum chemical hardness, which are the key parameters to find the molecular reactivity and radical scavenging ability. Overall, superheated steam extraction proved more beneficial, yielding more EO with a richer volatile composition and enhanced bioactivities compared with HD and SD. These results highlight the potential applications of *C. sinensis* EO as a natural source of bioactive compounds for the medicinal industries.

## Author Contributions

**Ahmed B. M. Ibrahim:** validation, software, project administration. **Nehha Nadeem:** investigation, writing – original draft. **Muhammad Adnan Ayub:** supervision, conceptualization, project administration, writing – review and editing. **Mohamed Fawzy Ramadan:** resources, data curation. **Khayala Mammadova:** methodology, validation. **Muhammad Ijaz:** formal analysis, methodology. **Mohamed A. Habib:** validation, methodology. **Nasrin Choobkar:** resources, project administration.

## Funding

This work was supported by Deanship of Scientific Research at Imam Mohammad Ibn Saud Islamic University (IMSIU) (grant number IMSIU‐DDRSP2602).

## Conflicts of Interest

The authors declare no conflicts of interest.

## Data Availability

The data that support the findings of this study are available from the corresponding author upon reasonable request.
